# Double-Barrel Anastomotic False Aortic Aneurysm

**DOI:** 10.7759/cureus.86734

**Published:** 2025-06-25

**Authors:** Keita Kamata, Mitsumasa Hata, Masashi Tanaka

**Affiliations:** 1 Department of Cardiovascular Surgery, Nihon University School of Medicine, Tokyo, JPN; 2 Department of Cardiovascular Surgery, Nihon University Hospital, Tokyo, JPN

**Keywords:** aortic aneurysm repair, aortic aneurysm surgery, distal tear, proximal tear, redo surgery, thoracic aortic aneurysm repair, thoracic aortic surgery

## Abstract

A 68-year-old woman, who had been well for two years after ascending aortic graft replacement, experienced sudden chest pain. Computed tomography showed a large false aortic aneurysm around the prosthetic graft. However, the patient was hemodynamically stable and did not have anemia. Urgent surgical procedure under hypothermic circulatory arrest revealed tears at both the proximal and distal anastomotic sites and blood circulating from the proximal (entry) to the distal (re-entry) tears underneath the pericardium. To our knowledge, the present case represents unreported pathology that could be of great interest to cardiologists and cardiac surgeons.

## Introduction

An aortic aneurysm is defined as a condition in which part of the wall of the aorta is circumferentially or locally enlarged or protruding. Generally, the diameter may be 30 mm in the chest and 20 mm in the abdomen, and when a portion of the wall is locally dilated (bulging or sac-like enlargement) or when the diameter exceeds 1.5 times the normal diameter and has expanded in a spindle-shaped manner, it is referred to as an “aneurysm.” Aneurysms are generally classified into true or pseudo-aneurysms. True aneurysms are characterized by a sac composed of the three layers of the aortic wall (intima, media, and adventitia). On the other hand, a pseudo-aneurysm lacks aortic wall components in its wall, and the new cavity formed outside the original aortic lumen is referred to as a pseudo-aneurysm. There are various causes of pseudo-aneurysms, but one cause is trauma to an artery, or, as in this case, an anastomotic failure following aortic surgery [1,2].

Identifying a false aortic aneurysm after replacement of the ascending aorta with an aortic prosthetic graft is a rare complication in this type of surgery. False aortic aneurysms show a marked increase in diameter and are associated with life-threatening complications, such as rupture, fistula formation, compression of adjacent organs, and thrombosis [3,4]. The etiology of such aneurysms is not clear, but they are considered to be due to infection or incomplete suturing of the anastomosis [5].

In this case, a patient underwent ascending aorta replacement for thoracic true aortic aneurysm and experienced mild chest pain two years later. The patient received massage therapy at an osteopathic clinic but did not improve, and thus she visited our hospital and was diagnosed with huge pseudo-aneurysms at both the proximal and distal anastomosis sites, requiring urgent re-operation. We present our experience in treating a double-barrel pseudo-aneurysm caused by rupture of both proximal and distal anastomoses.

This article was previously posted to the Authorea preprint server on December 08, 2022.

## Case presentation

**Figure 1:**
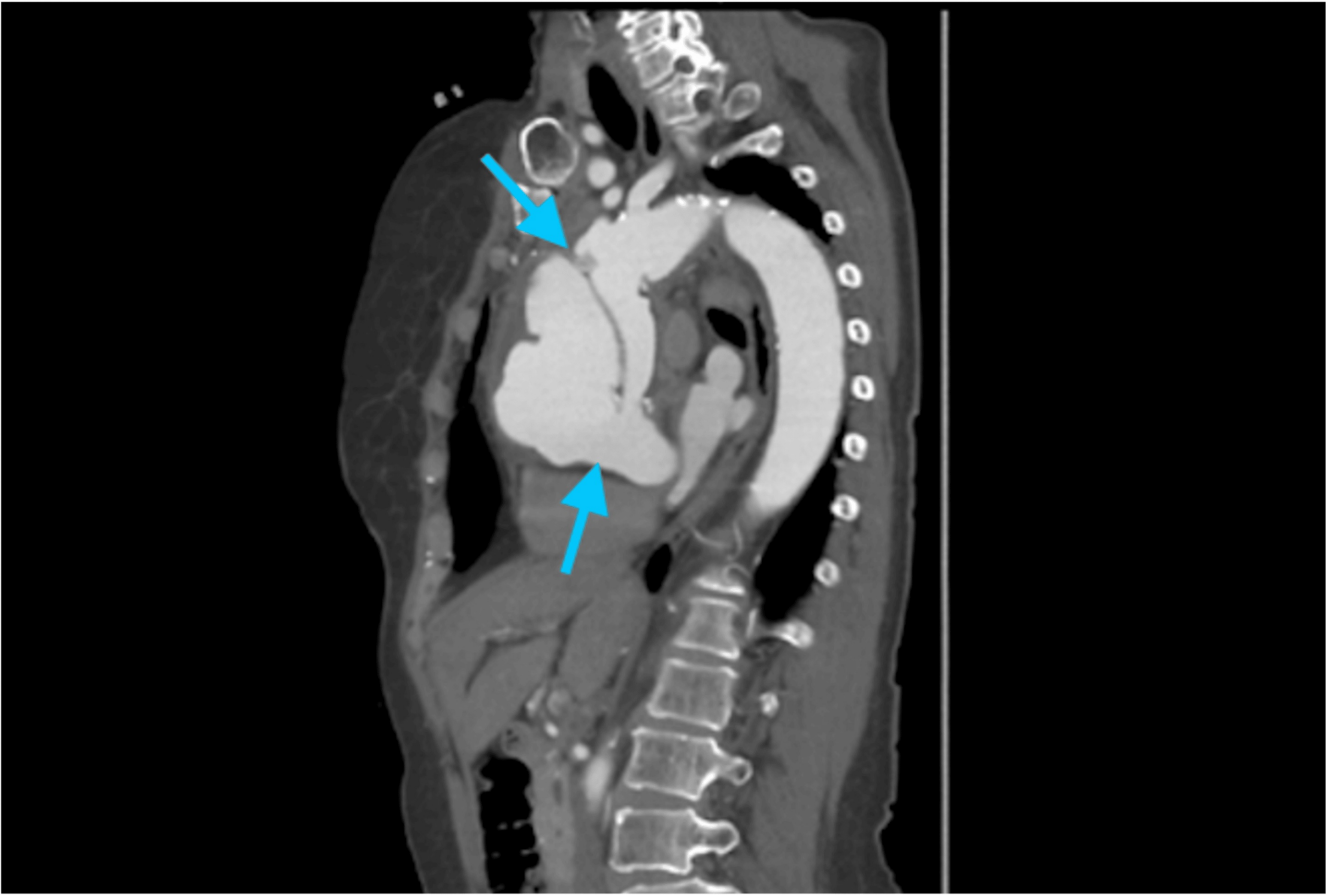
Sagittal CT image Proximal entry (lower blue arrow) and distal re-entry (upper blue arrow) into a false aneurysm of the ascending aorta following a previously placed ascending aortic graft.

**Figure 2:**
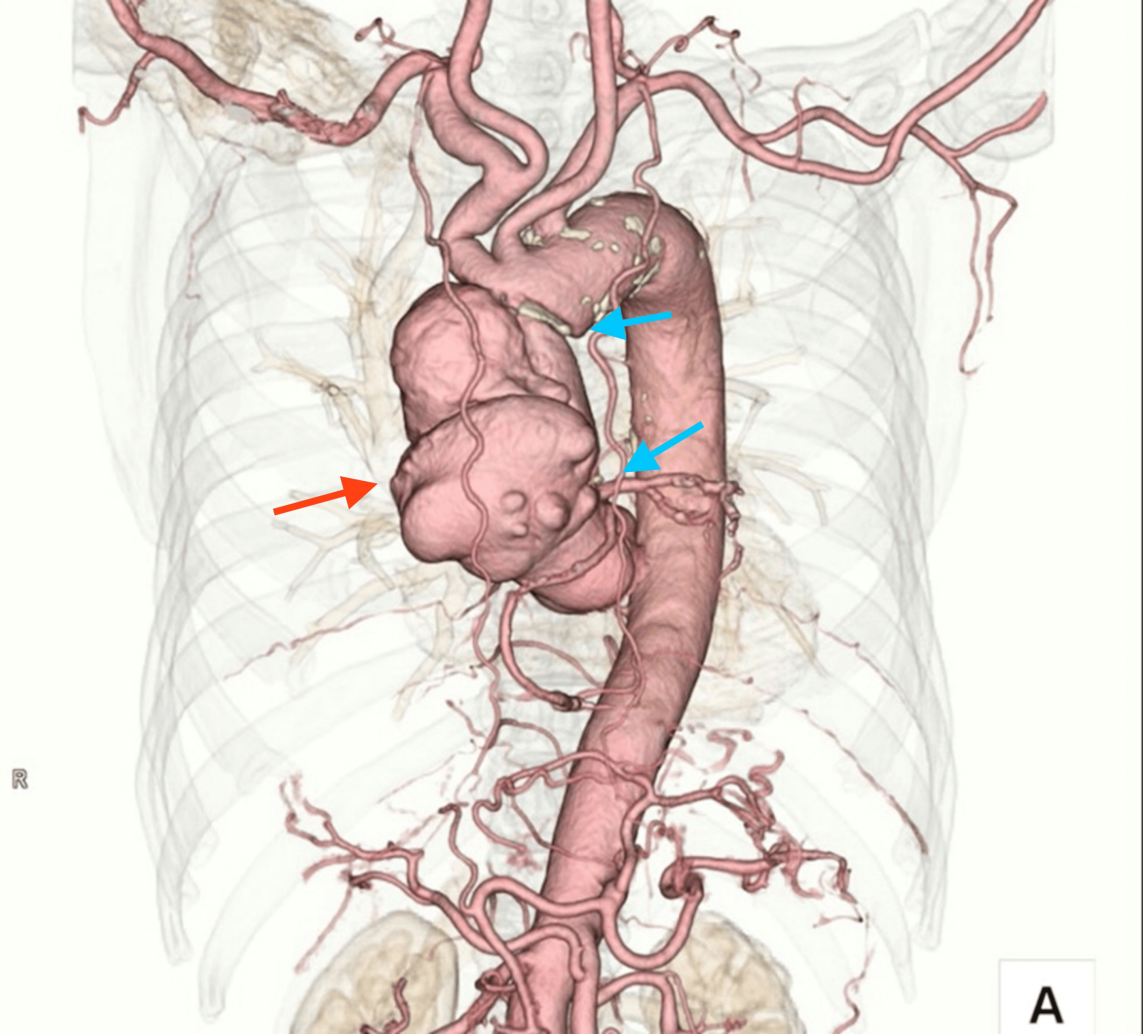
Three-dimensional CT aortography Anteriorly protruding false aortic aneurysm measuring 88 mm x 75 mm (red arrow) beginning at the proximal anastomosis (lower blue arrow) and ending at the distal anastomosis (upper blue arrow).

We present the case of a 68-year-old woman who had undergone aortic graft replacement for an ascending aortic aneurysm 2 years previously, January 2018, with no operative issues. However, one month before being treated at our facility, June 2020, she developed sudden chest pain. She did not seek medical advice, but a chest computed tomography (CT) examination performed at a regular outpatient visit showed a large false aneurysm of the ascending aorta around the implanted graft. The aneurysm was 88 mm × 75 mm in size and involved both the proximal and the distal graft suture lines (Figures 1, 2). It was covered by the pericardium that had been wrapped around the artificial graft from the previous operation, which was touching the sternum. A transthoracic echocardiogram showed that the false aneurysm was adjacent to the sinotubular junction and was around 80 mm in diameter. Cardiac function was preserved. A blood test showed no signs of infection with no evidence of anemia.

**Figure 3:**
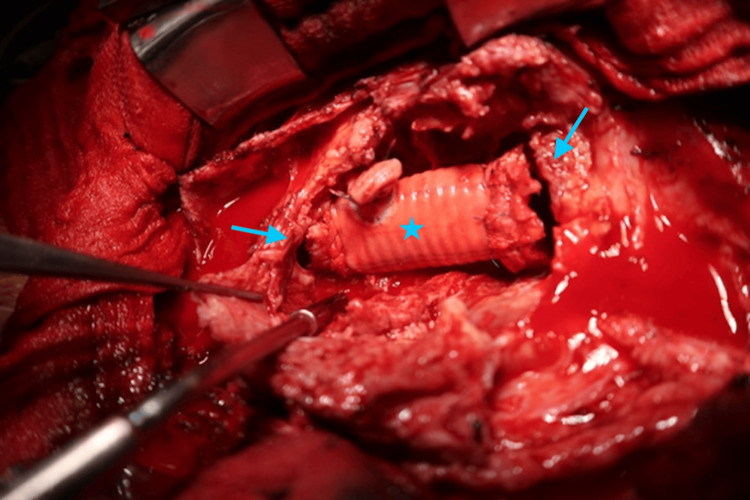
Intraoperative image. Note the pseudo-aneurysm wall opened, as well as the tears in the anastomosis sites of the proximal side (right arrow) and the distal side (left arrow) of the artificial graft (star) that were replaced in a previous surgery.

The patient was admitted to our hospital and underwent an urgent surgical procedure to repair the aneurysm. Initially, we placed the patient on cardiopulmonary bypass by cannulating the right femoral artery and right femoral vein. We used a median sternotomy approach. When we opened the pericardium, we found tears at both the proximal and the distal anastomotic sites of the previously placed the graft, which were massively bleeding (Figure 3). Immediately, cooling was started, and the proximal anastomosis was transected. Selective cardioplegia was performed, resulting in cardiac asystole. We arrested circulation at 25ºC and repaired the tear in the distal anastomosis with 4-0 polypropylene. The proximal side was then re-anastomosed with the previous graft. The patient was successfully removed from the cardiopulmonary bypass without major bleeding. After confirming hemostasis, we closed the chest and completed the operation.

## Discussion

Although the technology of stent grafting has advanced, open surgical procedures directed at replacement of the ascending aorta continue to be a major, established surgical technique for treating thoracic aortic aneurysms and dissection involving the ascending aorta. All of the known complications of this procedure, such as cerebral infarction, infections, and anastomotic insufficiency, can be life-threatening. In particular, rupture of the anastomosis is a serious event that generally leads to death.

False aortic aneurysm is a rare but serious complication of ascending aorta and/or aortic root prosthetic replacement. The incidence and risk factors are not clear. Mohammadi et al. reported that the incidence of false aortic aneurysm appears to be high in cases of aortic dissection, probably because of increased tissue fragility at the suture line of a graft replacement [4,5]. Other risk factors are known to increase the risk for the development of a false aortic aneurysm, including mediastinal infection after cardiac surgery and Takayasu’s arteritis [6-8]. Postoperative false aortic aneurysm is a late complication after aortic surgery. The time interval between the initial procedure and the occurrence of a false aortic aneurysm is variable but can be up to a maximum of 17 years [4,8].

The case presented here is noteworthy because of the underlying disease (true aneurysm), lack of infection, and pathological abnormalities, and in particular because it was a rare entry/re-entry type false aneurysm. We found another report of a false aneurysm due to tears at both the proximal and distal graft suture lines, but the aneurysm was not of the entry/re-entry type, and the original operation was due to an injury after a traffic accident, not a true aneurysm [5]. A previous case report described a pseudo-aortic aneurysm that formed a fistula to the right atrium [9], and other reports presented cases of a rupture at the site of aortotomy, proximal vein graft anastomosis, aortic cannulation, and distal anastomosis of ascending aortic graft replacement [10,11]. Pseudo-aneurysms caused by rupture of both proximal and distal anastomoses are rare, and cases such as this one, in which the patient survived without circulatory failure, underwent surgery, and was saved without complications, are extremely rare.

## Conclusions

Pseudo-aneurysms caused by anastomotic failure after aortic surgery are rare, but they are one of the complications that can lead to fatal outcomes. Although their clinical course varies, many cases require re-operation due to the possibility of rupture. When performing reoperation, the location of the entrance to the pseudoaneurysm is important because postoperative adhesion is expected. It is also essential to prepare a circulatory assist device in case of circulatory failure and consider the anastomosis method. Even in cases such as this where pseudo-aneurysms are present at both the proximal and distal anastomotic sites, we believe that active repair is desirable in consideration of the risk of rupture.
